# Mapping Temperature Distribution Generated by Photothermal Conversion in Graphene Film Using Er,Yb:NaYF_4_ Nanoparticles Prepared by Microwave-Assisted Solvothermal Method

**DOI:** 10.3389/fchem.2019.00088

**Published:** 2019-02-25

**Authors:** Oleksandr A. Savchuk, Joan J. Carvajal, Yolanda Cesteros, Pilar Salagre, Huu Dat Nguyen, Airan Rodenas, Jaume Massons, Magdalena Aguiló, Franscesc Díaz

**Affiliations:** ^1^Universitat Rovira i Virgili, Departament de Química Física i Inorgànica, Física i Cristal·lografia de Materials i Nanomaterials (FiCMA-FiCNA) and EMaS, Tarragona, Spain; ^2^Ultrafast Bio- and Nanophotonics Group, INL - International Iberian Nanotechnology Laboratory, Nanophotonics Department, Braga, Portugal; ^3^Universitat Rovira i Virgili, Departament de Química Física i Inorgànica, Catalytic Materials in Green Chemistry (GreenCat), Tarragona, Spain

**Keywords:** green synthesis, upconversion nanoparticles, graphene, nanothermometry, thermal mapping

## Abstract

This study analyzes the mapping of temperature distribution generated by graphene in a glass slide cover after illumination at 808 nm with a good thermal resolution. For this purpose, Er,Yb:NaYF_4_ nanoparticles prepared by a microwave-assisted solvothermal method were used as upconversion luminescent nanothermometers. By tuning the basic parameters of the synthesis procedure, such as the time and temperature of reaction and the concentration of ethanol and water, we were able to control the size and the crystalline phase of the nanoparticles, and to have the right conditions to obtain 100% of the β hexagonal phase, the most efficient spectroscopically. We observed that the thermal sensitivity that can be achieved with these particles is a function of the size of the nanoparticles and the crystalline phase in which they crystallize. We believe that, with suitable changes, these nanoparticles might be used in the future to map temperature gradients in living cells while maintaining a good thermal resolution.

## Introduction

Carbon-based materials have emerged as promising photothermal agents due to their wideband optical absorption that allows the absorption of light at various wavelengths leading to an efficient heat conversion (Han et al., 2018). The extraordinary properties of graphene propelled its application in many fields of science, including its use as photothermal conversion agent (Savchuk et al., 2016). The excellent performance of graphene as a photothermal conversion material has allowed its use in photothermal therapy, usually in combination with other functional materials with complementary properties (Zhu et al., 2010). However, we are not aware of the existence of studies on the thermal mapping of graphene films.

With the development of nanotechnology, the determination of temperature in a given system with submicrometric spatial resolution became possible. This has led to the development of a new subfield of thermometry, named nanothermometry, which studies the measurement of temperature at the nanoscale level (Lee and Kotov, 2007). Luminescence thermometry is considered to be one of the most promising non-contact techniques for temperature determination at the sub-micrometer and nanometer scale due to its very high spatial, thermal, and temporal resolutions, large measurement ranges and affordable costs (Brites et al., 2012; Jaque and Vetrone, 2012). A big number of materials has been studied for luminescence nanothermometry applications, including quantum dots (Maestro et al., 2010, 2014; Vlaskin et al., 2010; Benayas et al., 2015), organic dyes (Peterman et al., 2003; Steinegger et al., 2017; Xie et al., 2017), gold nanoparticles (Bomm et al., 2012; Shang et al., 2013), polymers (Graham et al., 2010; Okabe et al., 2012; Hannecart et al., 2015), and lanthanide doped materials (Cheng et al., 2013; Zheng et al., 2014; Cerón et al., 2015; Piñol et al., 2015; Zhu et al., 2016; Balabhadra et al., 2017). The different measurement techniques used, and based on changes in radiative lifetimes, intensity variations, spectral position shifting, and broadening of emission lines induced by temperature, have proved to be potential tools for temperature determination even in biosystems (Vetrone et al., 2010; Fischer et al., 2011; Du et al., 2014; Zhu et al., 2016; Li et al., 2017).

However, some of these materials attracted more attention because of their interesting advantages for luminescent nanothermometry. These materials are the Ln^3+^-doped upconversion nanoparticles (Ln^3+^-UCNPs). This kind of material absorbs light in the near-infrared (NIR) region of the electromagnetic spectrum, while emitting light in the visible range (Haase and Schäfer, 2011). Pumping in the NIR allows to overcome problems related to the background fluorescence arising from biological tissues, and the potential damage that ultraviolet (UV) light can generate in them (Diao et al., 2015), for instance. Finally, the use of NIR radiation also preserves the operative lifetime of the phosphors used in comparison with those illuminated with UV light, usually damaged by this radiation, that shortens their operational lifetimes (Rapaport et al., 2006).

Er^3+^ is the most used lanthanide ion for luminescence thermometry purposes in UCNPs because of its intense green emission that consists of two luminescence bands centered at 520 and 540 nm and assigned to the ^2^H_11/2_ → ^4^I_15/2_ and ^4^S_3/2_ → ^4^I_15/2_ radiative transitions, respectively. These two energy levels, from which the emission arises, are thermally coupled and, thus, the relative emission intensity of these two luminescence bands shows a strong temperature dependence (Vetrone et al., 2010; Fischer et al., 2011; Du et al., 2014; Zhu et al., 2016; Li et al., 2017). Among the potential crystalline matrices that can host Er^3+^, NaYF_4_ emerged as the most promising one (Krämer et al., 2004; Wang et al., 2010).

However, NaYF_4_ can crystallize in two polymorphic phases: the α-NaYF_4_ phase with $Fm\bar{3}m$ space group and the hexagonal β-NaYF_4_ phase with $P\frac{6_{3}}{m}$ space group (Wang et al., 2010). High reaction temperatures and long reaction times can induce the phase transformation from the metastable α phase to the thermodynamically stable β phase (Zhou et al., 2013). Thus, the synthesis of NaYF_4_ phosphors has been extensively studied, especially to explore the best conditions to obtain the pure β phase with a high production yield. Microwave-assisted hydrothermal synthesis emerged as an efficient method for the synthesis of monodispersed and highly luminescent NaYF_4_ nanoparticles (Wang and Nann, 2009), since it allows for a fast and uniform heating in an eco-friendly and energy-efficient way. However, those procedures still suffer from poor yields (Wang and Nann, 2009), production of mixtures of α and β phases (Mi et al., 2011), limited to microtubes (Chen et al., 2009; Tong et al., 2017), and nanowires (Wawrzynczyk et al., 2015), as well as micron-size particles (Som et al., 2016).

Here, we report the synthesis of Er,Yb:NaYF_4_ nanoparticles by a microwave-assisted solvothermal method which allowed us to obtain these nanoparticles in short times and at low temperatures. Furthermore, by tuning the basic parameters of the synthesis process, such as temperature and time of reaction, we succeeded in obtaining nanoparticles with different sizes and in isolating the different crystalline phases. We have been able to get the right conditions to obtain 100% of the pure β-NaYF_4_ phase with a production yield ranging from 64 to 98%. We also analyzed the temperature dependence of the luminescence of these nanoparticles. Nanoparticles with bigger sizes belonging to the hexagonal β phase showed a higher relative sensitivity than those with smaller sizes or those belonging to the cubic α phase. Finally, we used these nanoparticles to map the temperature distribution generated by the laser-induced heating of graphene deposited on a glass cover slide generated by a photothermal conversion process.

## Experimental Section

### Synthesis of Er,Yb:NaYF_4_ Nanoparticles

Yb (20 mol. %), Er (2 mol. %) co-doped NaYF_4_ nanoparticles were synthesized by a microwave-assisted solvothermal method. High purity Y_2_O_3_, Yb_2_O_3_, and Er_2_O_3_, analytical reagents, trisodium citrate (Na_3_C_6_H_5_O_7_), sodium fluoride (NaF), and ammonium fluoride (NH_4_F) were used as raw starting reagents. RE(NO_3_)_3_ (RE = Y, Yb, Er) were prepared by dissolving the corresponding RE_2_O_3_ in 10 ml of hot nitric acid (HNO_3_). After the evaporation of the nitric acid, 15–65.5 ml of ethanol (depending on the experiment) in which 8.1–24.7 g of trisodium citrate (depending on the experiment) were dissolved, were added and stirred for 60 min. In another vessel, 0.08–0.36 g of NaF and 0.45–3.62 g of NaH_4_F were dissolved in 7.5–60 ml of hot water, depending on the experiment. Then, the two solutions were mixed together. After a vigorous stirring for 2 h, the solution was transferred to a Teflon reactor with a total volume of 70 ml. This reactor was placed in a Milestone ETHOS-TOUCH CONTROL laboratory microwave autoclave, where it was maintained at a temperature between 393 and 453 K under continuous stirring during a period of time ranging from 3 to 6 h, depending on the experiment. Finally, the solution was cooled down naturally, and the precipitated nanoparticles were washed with ethanol and deionized water three times. Table 1 summarizes the synthesis conditions for the different experiments we performed.

**Table 1:** Synthesis conditions and main characteristics of the Er,Yb:NaYF_4_ nanoparticles obtained by the microwave-assisted solvothermal method.

**Temperature (K)**	**Time (h)**	**[Ethanol/Water] (Volume %)**	**Crystalline phase**	**Crystallite size (nm)**	**Shape**	**Production yield (%)**
453	6	70/30	β	63	Long rods	77
		60/40		74	Long rods	67
		50/50		55	Long rods	67
		40/60		68	Long rods	67
		30/70	α + β	48	Long rods	65
		20/80		42	Short rods	64
453	3	90/10	β	52	Spheres	98
		80/20		58	Spheres	97
		70/30		29	Irregular	97
		60/40		40	Spheres + short rods	96
		50/50		26	Irregular	86
		40/60		28	Irregular	71
423	3	90/10	α	22	Spheres	98
		80/20	β	128	Spheres	97
		70/30		60	Short rods	98
		60/40		57	Short rods	98
		50/50		37	short rods	75
		40/60		65	Short rods	69
393	3	80/20	β	62	Short rods	98
		70/30		55	Short rods	98
		60/40		72	Short rods	97
		50/50		72	Short rods	81
		40/60		58	Short rods	64

### Structural and Morphological Characterization

The crystalline structure of the obtained Er,Yb:NaYF_4_ nanoparticles was investigated by means of X-ray powder diffraction analysis using a Bruker-AXS D8-Discover diffractometer using Cu Kα radiation. The crystallite size was calculated with the data corresponding to all the reflections in the diffraction pattern using the Scherrer equation. The crystallite size is also listed in Table 1.

The morphology of the Er,Yb:NaYF_4_ nanoparticles obtained by the microwave-assisted solvothermal method was investigated using an environmental scanning electron microscope (ESEM) FEI Quanta 600 and a transmission electron microscope (TEM) JEOL 1011.

### Temperature-Dependent Luminescence Measurements

For temperature dependent luminescence measurements, the Er,Yb:NaYF_4_ nanoparticles were placed in a Linkam THMS 600 heating stage equipped with a BN disk that allowed an improved temperature distribution in the chamber. The nanoparticles were excited by a fiber-coupled diode laser emitting at 980 nm with a power of 10 mW, so that the contribution of the excitation laser to the heating of the nanoparticles is negligible. The laser beam was focused on the sample with a 40× microscope objective with a numerical aperture N.A. = 0.6, providing a spot size of around 100 μm on the sample. The visible emission arising from the nanoparticles was registered by the same microscope objective, and after passing a dichroic filter to eliminate the excitation radiation, it was fiber coupled to an AVANTES AVS-USB2000 spectrometer for the recording of the emission spectra. These spectra were recorded at temperatures between RT and 333 K.

### Thermal Mapping of Heat Transfer Through a Graphene-Coated Glass

Hundred microliters of a graphene solution in dymethylformamide (DMF) with a concentration of 1 mg ml^−1^ were deposited on a microscope slide cover glass with a thickness of 100 μm. The solvent was evaporated letting it dry at 353 K, generating a film with a thickness ranging between 700 nm and 1.2 μm, determined with a SENSOFAR PLμ 2300 confocal microscope. On the other side of the same microscope slide cover glass 100 μl of a dispersion of the Er,Yb:NaYF_4_ nanoparticles in ethanol with a concentration of 10 mg ml^−1^ was deposited. A fiber-coupled diode laser emitting at 808 nm with powers ranging from 20 to 200 mW, depending on experiments, was focused on the graphene-coated cover glass using a 60× microscope objective (N. A. = 0.7), generating a beam with a diameter of 9 μm on the sample. Graphene efficiently absorbs the 808 nm laser light and converts it into heat (Savchuk et al., 2016). The temperature generated was then determined through the analysis of the upconversion emission spectra of Er,Yb:NaYF_4_ nanoparticles.

## Results and Discussion

### Microwave-Assisted Solvothermal Synthesis of Er,Yb:NaYF_4_ Nanoparticles

**Scheme 1:**
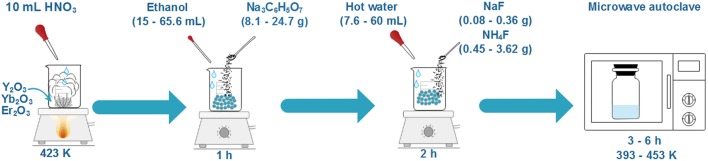
Schematic illustration of the microwave-assisted solvothermal method for Er,Yb:NaYF_4_ nanoparticles used.

Er,Yb:NaYF_4_ nanoparticles were synthesized with the microwave-assisted solvothermal method. The scheme of the synthesis process is shown in Scheme 1. Compared to other conventionally heated hydrothermal methods, microwave heating is a greener approach to the synthesis of materials, since it allows for a shortening of the reaction times, a reduction of the reaction temperature and thus, for a reduction of the energy consumption (Komarneni et al., 1992; Sánchez et al., 2013; Granados-Reyes et al., 2014). By changing the reaction temperature, the reaction time and the ethanol and water volumes, we obtained nanoparticles with different sizes and crystallizing in different crystalline phases, as listed in Table 1, identifying the conditions to obtain pure α- and β-NaYF_4_ nanoparticles. TEM and ESEM images of all the nanoparticles obtained are presented in Figure S1.

Since the β phase allows to obtain a higher up-conversion emission intensity, we focused our efforts in maximizing the production of this phase.

In the case of nanoparticles synthesized at 453 K for 6 h, the pure β-NaYF_4_ phase was obtained for solutions containing ethanol concentrations between 40 and 70%, while a mixture of the α and β phases (α phase with a lower concentration than the β phase) was obtained in solutions with a lower ethanol content. These solutions with a lower ethanol content were avoided in the remaining experiments. When the reaction time was reduced to 3 h, while keeping the reaction temperature at 453 K, the pure β phase was obtained for all the solutions analyzed. When the temperature was reduced to 423 K, the pure β phase was obtained for all the solutions analyzed, expect for the one formed by 90% ethanol and 10% water, in which we obtained the pure α phase. This solution composition was not used in the last experiment, in which the temperature was reduced to 393 K, while the reaction time was kept at 3 h. Under these conditions, we were able to obtain the pure β phase again for all the solutions analyzed.

The yields of production of the β phase were high in all cases, between 64 and 98%, as listed in Table 1. The production yields are higher for the samples prepared in solutions containing a higher ethanol content, and this does not seem to depend on the synthesis temperature. However, as the reaction time increased, the production yields decreased, which would indicate that the products formed tend to dissolve again with a long exposure to the solution.

The crystallite size of these nanoparticles was calculated using the Scherrer equation (Patterson, 1939). The results are listed in Table 1. Comparing the crystallite size of the nanoparticles obtained from solutions containing the same ethanol concentrations at different temperatures and reaction times, we observed that the smallest crystallite sizes were obtained for the nanoparticles synthesized at 453 K for 3 h, while, even when the temperature was reduced to 423 or 393 K, the crystallite size increased again.

If we take a look to the shape of the nanoparticles obtained, we can observe that at low synthesis temperatures we obtained short rods, but as the temperature increased these rods tended to transform toward more irregular or spherical shapes. However, the surfaces of these nanoparticles are not smooth, indicating that probably they are formed by the agglomeration of the smaller short rod units. Also, when the reaction time was increased from 3 to 6 h, sub-micrometric size particles exhibiting a long rod shape were obtained. Finally, comparing the shape of the nanoparticles obtained using different ethanol concentrations, we observed that as the ethanol concentration increased, the nanoparticles tended to show more irregular, spherical or long rod shapes, depending on the synthesis temperature and the reaction time used.

As explained in the Introduction of this article, microwave-assisted hydrothermal methods have been previously used for the synthesis of NaYF_4_ phosphors. Wang and Nann used sodium trifluoroacetate (TFA), yttrium-TFA, ytterbium-TFA, and erbium-TFA as initial reagents dissolved in oleic acid and 1-octadecene to obtain NaYF_4_ nanocrystals. They used a microwave-assisted process at 563 K for 5 min, obtaining cubic α-NaYF_4_ nanoparticles with very small sizes (11 nm in diameter) with a poor yield (Wang and Nann, 2009). Chen et al. (2009) used NaF, NH_4_HF_2_, and Y, Yb, and Tm nitrates as initial reagents dissolved in water to obtain NaYF_4_, adjusting the pH with HF, and heating the solution at 453 K for 4 h in a microwave synthesizer. They obtained β-NaYF_4_ microtubes, 0.5 μm in diameter and 2–3 μm long. Similar microtubes were obtained by Tong et al. (2017) using a similar methodology. Later, Mi et al. (2011) used rare earth acetates, such as NH_4_F and NaCl, and fluorine and sodium sources, respectively, dissolved in a mixture of water and ethylene glycol, and treated thermally in a microwave autoclave at 433 K for 1 h. Under these conditions they obtained a mixture of the α and β phases with average sizes of around 40 nm. More recently, Wawrzynczyk et al. (2015), using a microwave flash-heating method, obtained NaYF_4_ nanowires 1.5 mm long and 100 nm in diameter. On the other hand, Som et al. (2016) prepared Er,Yb:NaYF_4_ phosphors using rare earth nitrates and NaF dissolved in water, and adding ethylenediamine tetra acetic acid (EDTA) as chelating agent. They adjusted the pH of the solution with NaOH in a microwave digestion unit at 453 K for different time periods extending from 10 min to 2 h. The samples obtained at shorter times crystallized in the α metastable phase with small amounts of YF_3_, indicating that the reaction was not complete. For intermediate times they obtained mixtures of the α and β phases, while the samples obtained at longer times exhibited only the pure β-NaYF_4_ phase with a hexagonal prism shape, with a mean diameter of 1.0 μm and a length of 5.5 μm. More recently, Palo and co-workers obtained NaYF_4_ nanoparticles using a microwave-assisted solvothermal method at relatively low temperatures (413–458 K) and long times (4–12 h) (Palo et al., 2016). However, the resulting nanoparticles are a mixture of the two phases with different morphologies (spherical, large cubes, and rods). Wang et al., in their study, synthesized β-NaYF_4_ nanoparticles uniform in size and with s strong fluorescence using a solvothermal method. The nanoparticles prepared using the synthesis optimal conditions (453 K, 24 h, and 5 ml of oleic acid) were used as fluorescent labels for fingerprint applications (Wang et al., 2015).

Thus, when comparing the results previously reported with the ones we obtained, we can conclude that the microwave-assisted solvothermal method we developed allows obtaining pure β-NaYF_4_ nanoparticles with smaller sizes and at lower temperatures and shorter reaction times. Also, we avoided the appearance of microtubes or nanowires. Furthermore, the synthesis procedure we present here avoids the use of organic ligands like oleic acid (Wang and Nann, 2009; Wang et al., 2015) or EDTA (Som et al., 2016), which confer a hydrophobic character to the nanoparticles obtained and are difficult to eliminate at the end of the reaction. With our synthesis procedure, the production yields obtained ranged from 64 to 98%, as can be seen in Table 1. Thus, the methodology presented here shows several advantages over the technologies previously reported in the literature, such as the use of lower temperatures, the production of pure α and β phases, the possibility to avoid mixtures of both phases, or the use of cheaper solvents avoiding organic ligands that have to be removed at the end of the synthesis process. Furthermore, the nanoparticles obtained are already dispersible in water and biological compatible fluids, without requiring any post-growth treatment, which is a clear advantage when one expects to use such nanoparticles for biological applications.

**Figure 1:**
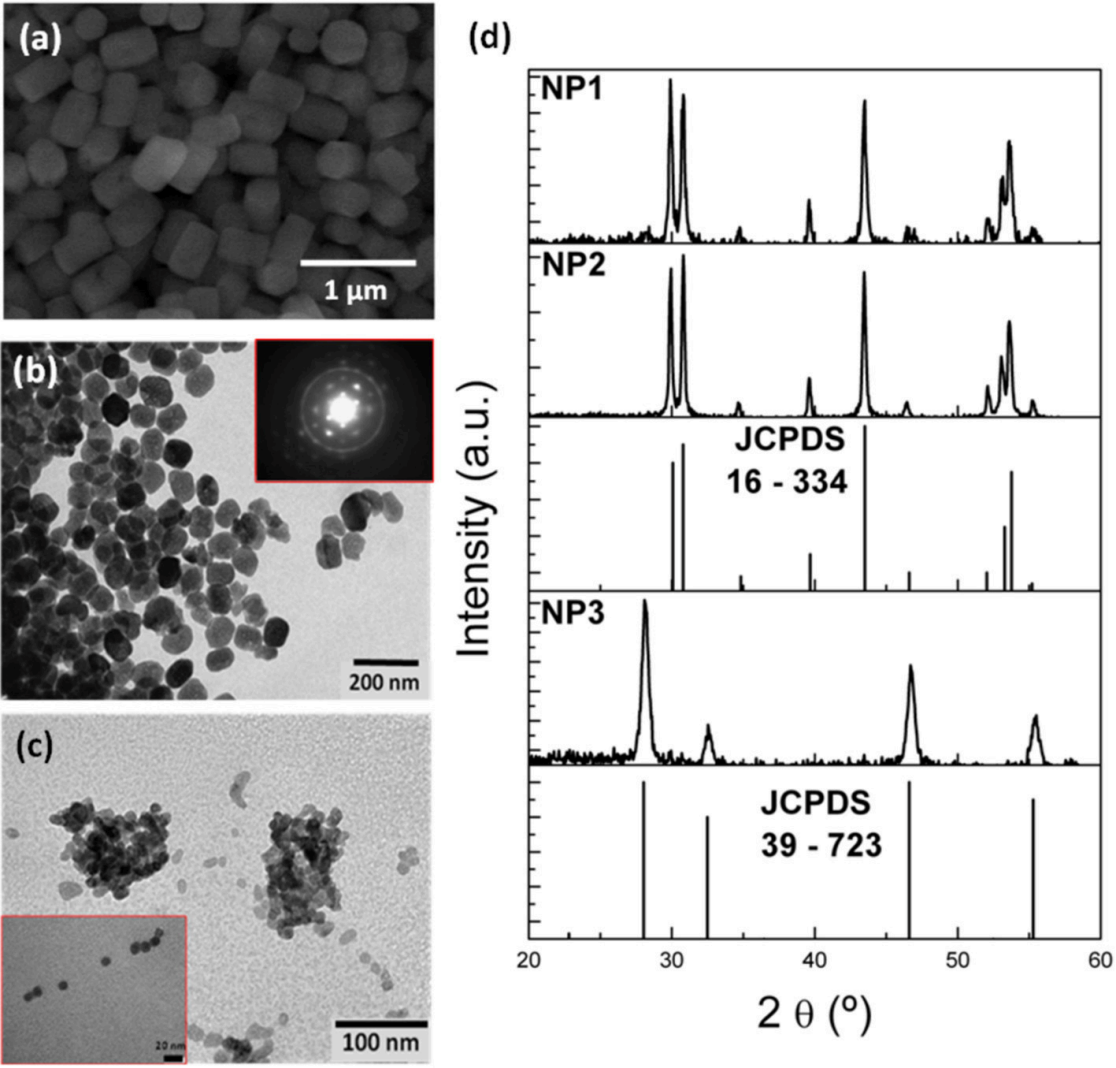
**(a)** ESEM image of the nanoparticles obtained in the NP1 sample. TEM images of the nanoparticles obtained in the **(b)** NP2 and **(c)** NP3 samples. **(d)** X-ray diffraction patterns of the samples, together with the reference diffraction patterns for the α- and β-NaYF_4_ crystalline phases.

We selected three representative sets of nanoparticles from the experiments performed: NP1, synthesized at 453 K for 6 h with an ethanol/water volume ratio 70/30; NP2, synthesized at 453 K for 3 h with an ethanol/water volume ratio 80/20; and NP3, synthesized at 423 K for 3 h with an ethanol/water volume ratio 90/10. An ESEM image of the NP1 nanoparticles is shown in Figure 1a. They show a prismatic shape with a hexagonal base, 300 nm in diameter and 600 nm long. This is the morphology we labeled as long rods in Table 1. Instead, samples NP2 and NP3 show nanoparticles with an almost spherical shape with average sizes of 50–70 and 20 nm, respectively, as observed in the TEM pictures shown in Figures 1b,c. They correspond to the morphologies labeled as irregular and spherical, respectively, in Table 1. The sizes of the nanoparticles corresponding to NP2 and NP3 samples, determined from the TEM pictures, match the crystallite sizes determined using the Scherrer equation. In the case of the NP1 sample, however, a large discrepancy was observed. This can be due to the fact that the size of these sub-micrometer particles is at the limit of the validity range for size determination using this equation, established at around 500 nm, and also to the fact that the Scherrer equation only accounts for one of the dimensions of the particles, that in this case were constituted by hexagonal prisms (Muniz et al., 2016). Figure 1d shows the X-ray diffraction (XRD) patterns recorded for these samples. We observed that samples NP1 and NP2 crystallize in the hexagonal system, with space group P6_3_/m corresponding to the β-NaYF_4_ phase, as confirmed by the comparison with the JCPDS reference pattern 16–334, also included in the figure. In contrast, the nanoparticles in sample NP3, which were synthesized at a lower temperature and for a shorter time, crystallize in the cubic system, with space group Fm3m corresponding to the α-NaYF_4_ phase, as confirmed by the comparison with the JCPDS reference pattern 39–723, also included in the figure. Additionally, we confirmed that the β-NaYF_4_ nanoparticles obtained with this procedure were highly crystalline, as confirmed by the SAED pattern obtained by electron diffraction of one of these nanoparticles included in the inset of Figure 1b.

### Temperature-Dependent Luminescence Measurements

**Figure 2:**
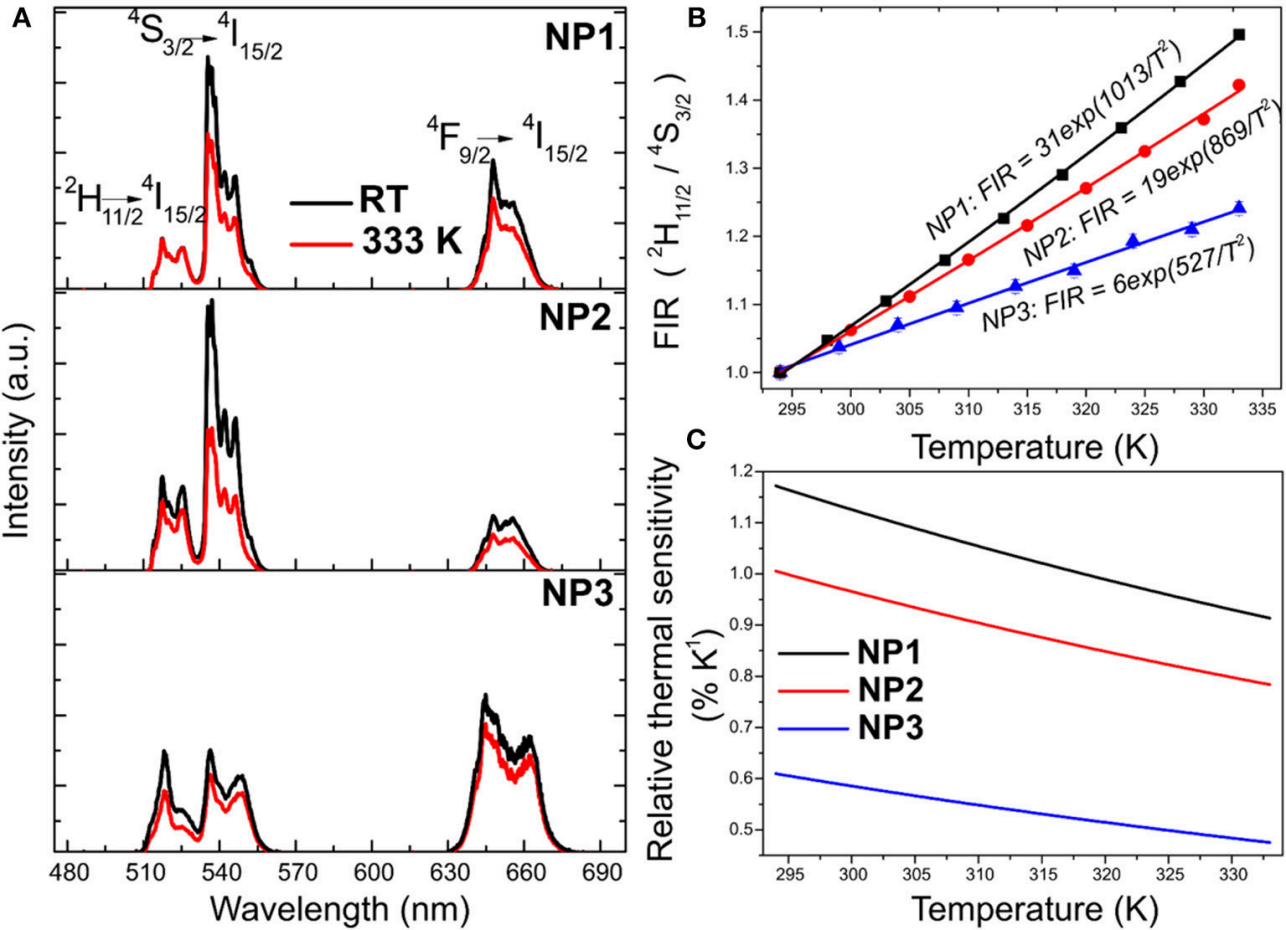
**(A)** Upconversion emission spectra at room temperature (black) and 333 K (red) for NP1, NP2, and NP3 samples. **(B)** Normalized FIR as a function of temperature for NP1 (black), NP2 (red) and NP3 (blue) samples with the corresponding fitting following a Bolzmann thermal distribution. **(C)** Relative thermal sensitivity as a function of temperature for the three samples [NP1 (black), NP2 (red), and NP3 (blue)].

The emission spectra of these nanoparticles after excitation at 980 nm are shown at room temperature and at 333 K in Figure 2. These spectra consist of two green bands centered at 520 and 540 nm, assigned to the ^2^H_11/2_ → ^4^I_15/2_ and ^4^S_3/2_ → ^4^I_15/2_ transitions of the Er^3+^ ion, respectively. An additional red band was observed at 630–670 nm, assigned to the ^4^F_9/2_ → ^4^I_15/2_ transition of Er^3+^. While this red band shows a lower intensity than the green bands in samples NP1 and NP2, it is the main band in sample NP3, associated with the different crystalline phases of these nanoparticles.

The pathways for the generation of these emission lines through an upconversion process are well-known and have been previously used for luminescence thermometric applications (Vetrone et al., 2010; Dong et al., 2014; Jiang et al., 2014). Excitation at 980 nm promotes electrons of Yb^3+^ from the ^2^F_7/2_ fundamental state to the ^2^F_5/2_ excited state, from where an energy transfer process to Er^3+^ occurs, populating its ^4^I_11/2_ energy level. A second energy transfer process from Yb^3+^ promotes the electrons of Er^3+^ from the ^4^I_11/2_ to the ^4^F_7/2_ level. Then, non-radiative relaxation processes populate the ^2^H_11/2_ and ^4^S_3/2_ states, which results in the ^2^H_11/2_ → ^4^I_15/2_ and ^4^S_3/2_ → ^4^I_15/2_ radiative decays, emitting photons at 520 and 540 nm, respectively. The ^4^F_9/2_ energy level is populated by a new energy transfer process from Yb^3+^ to Er^3+^ that occurs after a non-radiative relaxation process from the ^4^I_11/2_ to the ^4^I_13/2_ energy level.

As observed in Figure 2A, the intensity of the visible emissions decreased when the temperature increased for all samples.

The fluorescence intensity ratio (FIR) technique has proven to be an efficient tool to evaluate the luminescence thermometry properties of the green emissions generated by Er^3+^ in several materials, including Er,Yb:NaYF_4_ nanoparticles (Vetrone et al., 2010; Dong et al., 2014; Jiang et al., 2014). This technique is based on the comparison of the intensities of two emission bands arising from two closely spaced energy levels, the ^4^S_3/2_ and ^2^H_11/2_ levels of Er^3+^ in this case, whose populations are in thermal equilibrium governed by a Boltzmann distribution. Figure 2B shows the evolution of the FIR with temperature. FIR can be calculated based on a Boltzmann distribution equation defined as (Wade et al., 2003):

(1) $$\begin{array}{l} FIR\left(\frac{I_{520}}{I_{540}}\right)=\frac{g_{1}\gamma_{1}\sigma_{1}}{g_{2}\gamma_{2}\sigma_{2}}e^{\left(-\frac{\Delta E}{kT}\right)} \end{array}$$

where *I*_520_ and *I*_540_ are the intensities of the emission bands located at 520 and 540 nm, respectively, *g*_i_ are the degeneracy of levels, γ_i_ are the spontaneous emission rates, σ_i_ are the absorption rates, Δ*E* is the energy difference between the two thermally coupled energy levels involved in the radiative transitions, *k* is the Boltzmann constant, and *T* is the absolute temperature.

Experimental points for the different samples were fitted to Equation 1, and the obtained expressions are included in Figure 2B. The nanoparticles that show the highest slope are those corresponding to the NP1 sample.

The qualitative performance of these nanoparticles to sense small changes in temperature was obtained by calculating the relative thermal sensitivity as the first derivative of FIR with respect to temperature divided by the FIR (Brites et al., 2012). The relative thermal sensitivities of the analyzed samples are included in Figure 2C. The NP1 sample, with nanoparticles exhibiting bigger sizes, possesses the highest thermal sensitivity with a maximum of around 1.2% K^−1^ at 333 K. The thermal sensitivity of the NP2 sample is smaller than that of NP1. Thus, based on the presented results we can conclude that at the sub-micron and nanoscale, smaller β-Er,Yb:NaYF_4_ nanoparticles would show smaller thermal sensitivities. This can be explained by the presence of a higher concentration of luminescent active ions close to the surface of smaller nanoparticles due to their higher surface to volume ratio. These ions would interact with the ligands attached to the surface of the nanoparticles, leading to concentration quenching processes that would affect negatively their thermal sensitivity. This might seem at odds with the data reported by Dong et al. (2014), since they reported that β-Er,Yb:NaYF_4_ particles with smaller sizes presented a higher thermal sensitivity. However, they analyzed micron-size particles, while the nanoparticles here presented lay in the sub-micron and nanoscale. Nevertheless, the thermal sensitivity of the β-Er,Yb:NaYF_4_ nanoparticles synthesized by this microwave-assisted solvothermal method is similar, or even slightly higher, than that reported for the same nanoparticles synthesized with other methods, in the range 0.21–1.24% K^−1^ (Vetrone et al., 2010; Fischer et al., 2011; Wu et al., 2011; Sedlmeier et al., 2012; Dong et al., 2014). Finally, α-Er,Yb:NaYF_4_ nanoparticles in the NP3 sample showed the smallest thermal sensitivity. This is not surprising since the luminescence efficiency of the α-phase has been reported to be smaller than that of the β-phase, despite its thermal efficiency for luminescence thermometry has not been reported before. The thermal resolution that can be achieved with these nanoparticles, calculated by dividing the precision of the detection system (0.5% in our case) by the relative thermal sensitivity, is presented in Figure S2.

### Temperature Distribution Mapping in Graphene by Luminescence Thermometry

**Figure 3:**
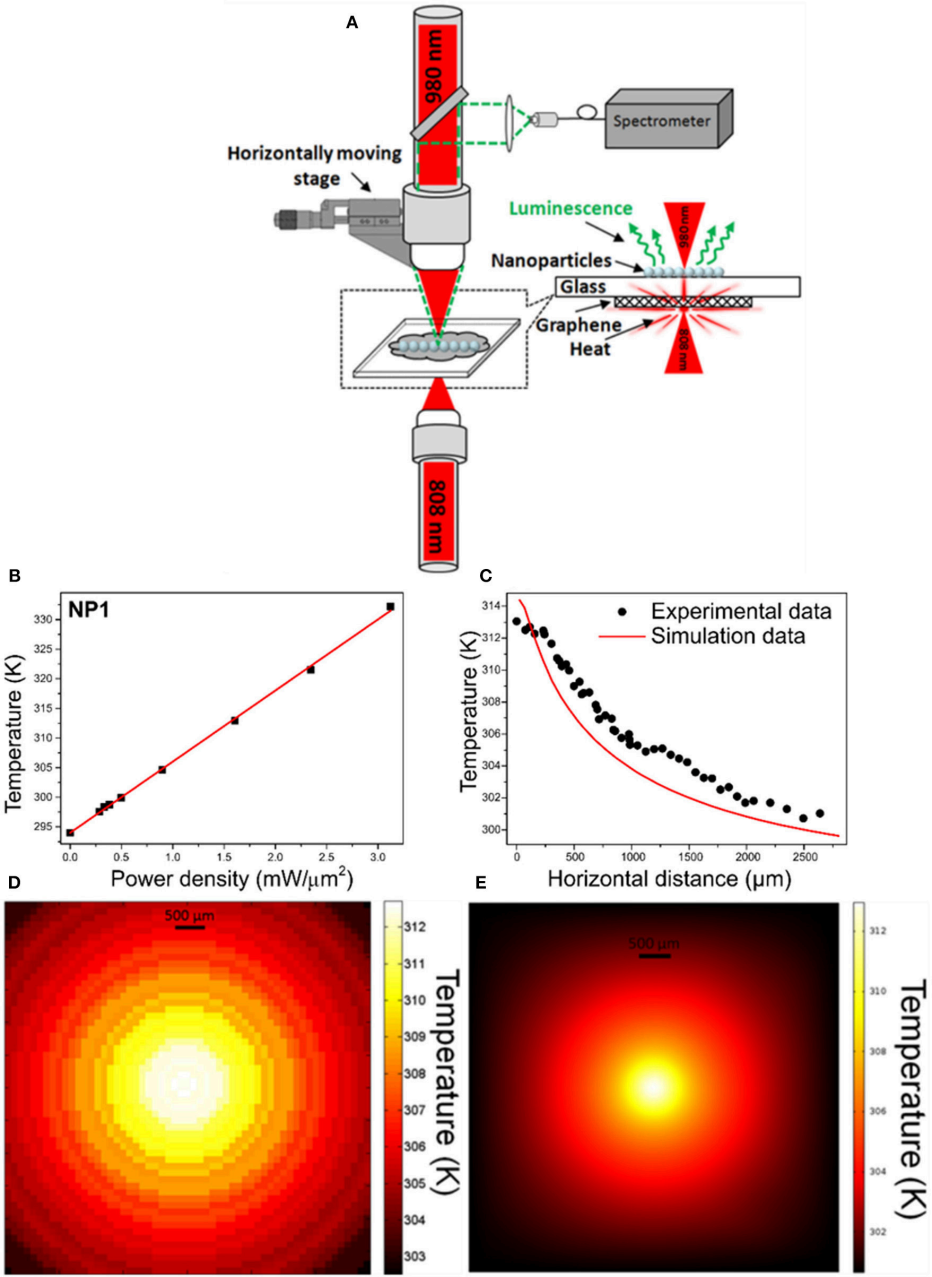
**(A)** Scheme of the setup used to map the temperature distribution generated by graphene when illuminated with a 808 nm laser on a microscope slide cover glass. **(B)** Temperature achieved on the surface of the graphene-coated glass as a function of the power of the irradiating laser at 808 nm. **(C)** Temperature distribution generated by graphene on the slide cover glass determined from the luminescence spectra of the Er,Yb:NaYF_4_ nanoparticles and comparison with the temperature distribution calculated from a heat transfer model in a 3D geometry. 2D temperature contour map generated from the data obtained **(D)** experimentally, and **(E)** with the 3D model.

In order to prove the potentiality of the Er,Yb:NaYF_4_ nanoparticles synthesized through the microwave-assisted solvothermal method, we used them to map the temperature distribution generated on a glass coated with graphene when illuminated with a laser beam. In order to do this, we took a 100 μm thick microscope slide cover glass and we coated it with graphene flakes on one side, generating a continuous film with a thickness varying from 700 nm to 1.2 μm, and Er,Yb:NaYF_4_ nanoparticles (corresponding to the NP1 sample) on the other side. Two spatially overlapped lasers were focused on both sides of the slide cover glass. A fiber-coupled diode laser emitting at 808 nm with a beam diameter of 9 μm was focused on the face of the cover glass coated with graphene. Graphene efficiently absorbs the 808 nm laser light and converts it into heat (Savchuk et al., 2016). The propagation of the heat generated by graphene on the opposite side of the cover glass was measured by determining the temperature through the spectra generated by the Er,Yb:NaYF_4_ nanoparticles when excited with a fiber-coupled diode laser emitting at 980 nm with a beam diameter of 10 μm and by using the calibration curve shown in Figure 2B. The scheme of this experimental setup is shown in Figure 3A.

First, we measured the temperature that can be achieved in the system when the power of the 808 nm laser focused on graphene was increased. The results are shown in Figure 3B. We observed that the temperature increased linearly with the power of the laser. Based on these results, we decided to set the power of the laser at 100 mW, which allows for the temperature to achieve ~312 K on the opposite side of the glass in order to get a constant heating and generate a thermal gradient high enough on the surface of the cover glass coated with the Er,Yb:NaYF_4_ nanoparticles to be easily detected with them. The 980 nm excitation laser with a fixed excitation power of 100 mW was attached to a motorized stage that allowed to scan the sample below the microscope setup. We recorded the spectra of the Er,Yb:NaYF_4_ nanoparticles in 50 μm steps. It took 60 s to record each spectrum, and the total scan duration was of 1 h. We did not observe any reduction of the emission intensity during the excitation with the 980 nm laser at a particular position of the setup, and this could be attributed to a possible heating of the nanoparticles. Thus, the power of the excitation laser we used was low enough to avoid the introduction of any artifact on the mapping of the temperature distribution in graphene. Figure 3C shows the temperature profile measured from the heating spot (corresponding to the 0 position) to the external part of the film along the horizontal direction and Figure 3D represents 2D temperature contour map of experimental data. The temperature decreased slowly along this direction, dropping down from 313.0 to 300.5 K. We were able to detect temperature changes as small as 0.2 K, smaller than the one predicted by the thermal resolution calculated from the thermal sensitivity (see Figure S2).

To contrast these results, the temperature distribution on the glass substrate was numerically analyzed by a finite element method (FEM) based on the commercial software COMSOL Multiphysics. The simulation was performed in a three-dimensional (3D) geometry using the heat transfer model for a steady-state study. To model the experiment, the computational domains were defined by two attached blocks which represent the glass substrate and the deposited graphene layer. The glass block used had an area of 10 × 10 mm^2^, and a height of 100 μm. The graphene block was constructed with an area of 10 × 10 mm^2^, and a height of 1 μm, based on the mean thickness value determined for the graphene film. In order to assign the laser beam as a heating source, a cylindrical domain (9 μm diameter, 1 μm height) was additionally created in the middle of the graphene domain corresponding to the waist of the pumping laser. A triangular mesh was defined with a well-defined size (minimum 1 μm) within the small domain (the cylindrical heat source domain) and a coarser size (maximum 1 mm) within the big domains (glass and graphene domains). To solve the heat transfer problem in the stationary state, the following thermal conductivity values of the materials were assigned to the corresponding domains: *k* = 3,100 W m^−1^ K^−1^ for graphene (Pop et al., 2012), and *k* = 1.38 W m^−1^ K^−1^ for the silica glass (Bansal and Doremus, 2013). The heating source was introduced to the cylindrical graphene domain with an overall heat transfer rate of 100 mW. The boundary conditions were specified with a convective heat flux in all the boundaries in which a convection coefficient (h) was determined from the best fit to the measured temperature distribution. The value of h obtained was 80 W/m^2^ K, which is reasonable for the natural convection conditions taking place in the experiment.

Figure 3E shows the heat gradient calculated within the approximate range of 300 and 314 K, which closely matches the experimental results. The exponential decay trend deduced from this model is followed by the temperature profile determined experimentally with the luminescent nanoparticles. The small discrepancies that can be observed between the two profiles might be attributed to the fact that the thickness of the deposited graphene layer was not homogeneous, and also to the grain boundaries generated in this kind of films, as well as to the poor bonding between the graphene film and the glass slide, parameters that are critical in a heat transfer process, while these factors were idealized in the simulation. Also, in the model, the thermal conductivity of graphene was assumed to be isotropic whereas this might have an anisotropic behavior. Finally, by switching on the 980 nm emitting laser only during the time required to record the luminescence in each point, it would produce even more precise values of temperature. Nevertheless, and despite these considerations, the thermal gradient we measured using the Er,Yb:NaYF_4_ nanoparticles followed the same trend than the simulated one.

As previously reported in the literature (Vetrone et al., 2010; Fischer et al., 2011), we believe that, by internalizing these luminescent nanoparticles on living cells, with the required suitable chemical functionalization of their surfaces as prior steps, thermal contour maps would also be obtained by *in vitro* procedures with the same thermal sensitivity, allowing for the illustration of the internal temperature gradients in the cells.

## Conclusion

In summary, we synthesized Er,Yb:NaYF_4_ nanoparticles with a microwave-assisted solvothermal method at lower temperatures and reaction times than previously reported methods. Furthermore, this synthesis method allowed to produce the α- and β phases of Er,Yb:NaYF_4_ separately, avoiding the production of mixtures and avoiding also the use of organic solvents and ligands not miscible with water or difficult to eliminate after the reaction. An additional advantage of this synthesis method is that the produced nanoparticles are hydrophilic and can be dispersed in water or other biological compatible fluids without requiring any post-growth chemical functionalization procedure.

We analyzed the temperature dependence of the upconversion emission of these nanoparticles for their use as luminescent nanothermometers. We observed that nanoparticles with bigger sizes possess higher thermal sensitivities. Their thermal sensing capabilities were proved by determining the temperature distribution induced by the light to heat conversion generated by a graphene layer deposited on a microscope slide cover glass when illuminated with a laser emitting at 808 nm. With this experiment we wanted to show that these nanoparticles can be used to monitor the temperature increase generated by graphene and derivatives when illuminated with a laser, one of the most promising techniques nowadays for tumor treatment by hyperthermia. We believe that by using the suitable chemical functionalization steps, these nanoparticles can be internalized in living cells to visualize their internal temperature distribution maps through *in vitro* procedures with a similar thermal resolution.

## Author Contributions

All authors listed have made a substantial, direct and intellectual contribution to the work, and approved it for publication.

### Conflict of Interest Statement

The authors declare that the research was conducted in the absence of any commercial or financial relationships that could be construed as a potential conflict of interest.
