# Supplementary material for: Mapping Temperature Distribution Generated by Photothermal Conversion in Graphene Film Using Er,Yb:NaYF4 Nanoparticles Prepared by Microwave-Assisted Solvothermal Method
Source: Front Chem. 2019 Feb 25;7:88. doi: 10.3389/fchem.2019.00088 (PMC6397865; doi:10.3389/fchem.2019.00088)
Supplement: Supplementary file 1 [file Data_Sheet_1.PDF]

Supplementary Information

Mapping temperature distribution generated by photothermal conversion in graphene film using Er,Yb:$NaYF_4$ nanoparticles prepared by microwave-assisted solvothermal method

Oleksandr A. Savchuk*, Joan J. Carvajal, Yolanda Cesteros, Pilar Salagre, Huu Dat Nguyen, Airan Rodenas, Jaume Massons, Magdalena Aguiló, Franscesc Díaz

*** Correspondence:** Corresponding Author: oleksandr.savchuk@inl.int

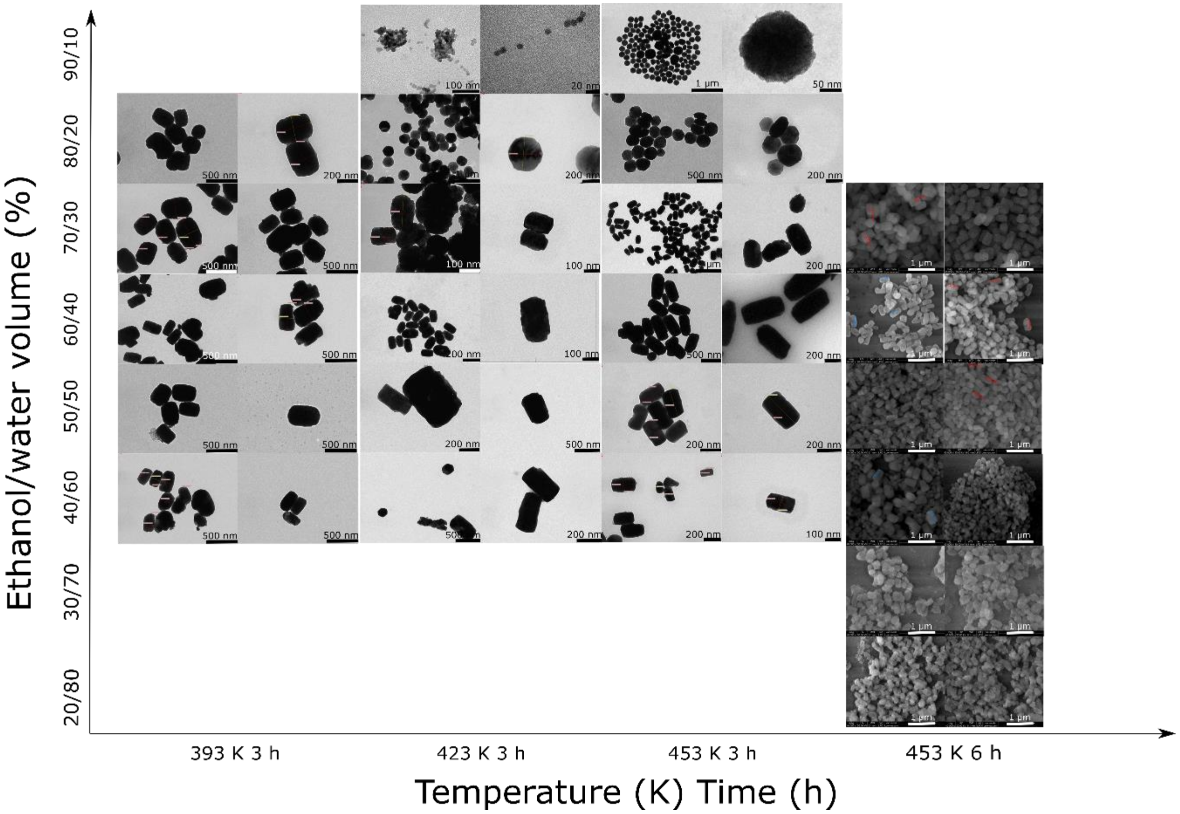

Figure S1| TEM and ESEM images of all set of prepared nanoparticles at different reaction time and ratio of ethanol/water volume.

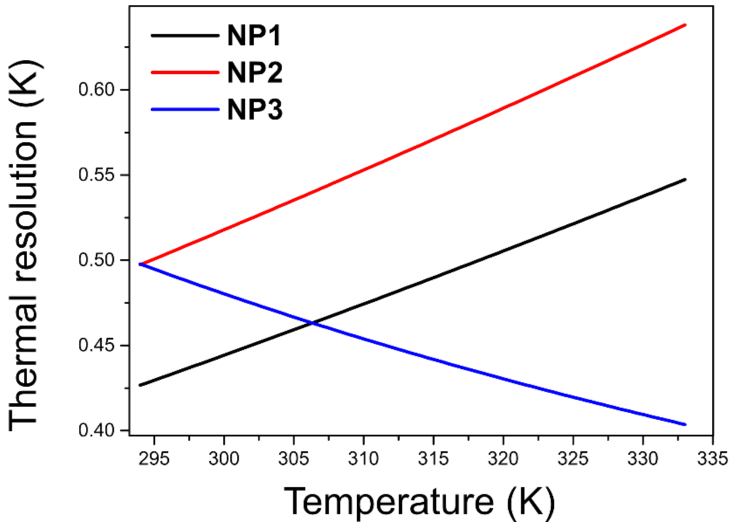

Figure S2| Thermal resolution calculated for NP1, NP2 and NP3 samples.
